# Respiratory illness healthcare-seeking behavior assessment in the Lao People’s Democratic Republic (Laos)

**DOI:** 10.1186/1471-2458-13-444

**Published:** 2013-05-04

**Authors:** Mayfong Mayxay, Visanou Hansana, Bouachanh Sengphilom, Latsamy Oulay, Vatsana Thammavongsa, Vatsana Somphet, Chansathit Taykeophithoune, Soudavanh Nathavong, Johnly Phanthady, Kongmany Chareunvong, Phetsavanh Chanthavilay, Vanphanom Sychareun

**Affiliations:** 1 Faculty of Postgraduate Studies, University of Health Sciences, Vientiane, Lao PDR; 2 Lao-Oxford-Mahosot Hospital-Wellcome Trust Research Unit (LOMWRU), Microbiology Laboratory, Mahosot Hospital, Vientiane, Lao PDR; 3 Centre for Clinical Vaccinology and Tropical Medicine, Nuffield Department of Clinical Medicine, Churchill Hospital, University of Oxford, Oxford, UK

**Keywords:** Respiratory illness, Healthcare-seeking behaviour, Laos

## Abstract

**Background:**

Respiratory illness (RI) remains a public health problem in Laos, but little is known about the overall burden and people’s healthcare-seeking behavior for RI. Understanding the burden of RI and community patterns of healthcare-seeking behavior would provide better guidance for Lao public health program and policy planners to improve RI public health practice, surveillance systems, and prevention strategies.

**Methods:**

A quantitative and qualitative survey was conducted in 14 randomly selected villages of two purposively selected peri-urban and two rural provinces in Laos. A pre-designed and pre-tested questionnaire was used to collect information on RI in household members (defined as new fever with cough and/or sore-throat in the absence of other diagnoses during the preceding 30 days) from all heads of household in each village. Sixteen focus group discussions were conducted to obtain more information to support the quantitative survey.

**Results:**

Among 1,751 households (9,114 people) studied, 3.5% (317/9,114) had experienced RI (fever, cough, and/or sore-throat) in the 30 days before the survey [6.2% in rural and 2.4% in peri-urban areas (p<0.001)]. The percentage of RI among persons aged ≥15 years was 2.7%, 3.7% for those aged 5 – 14 years, and 8.2% for children < 5 years (p<0.001). Of all sick persons, 71% sought treatment [94% in peri-urban and 48% in rural areas (p<0.001)] and 31.5% of them self-medicated [55.5% in peri-urban and 29% in rural areas (p<0.001)]. Sick people in peri-urban areas preferred to chose private clinics and pharmacies as their first treatment option while in rural areas they frequently consulted with village health volunteers and visited health centres as their first choice. The qualitative study suggests that distance, costs of care, and service availability are the most important determinants of seeking healthcare.

**Conclusions:**

The RI burden and healthcare-seeking behavior are different between rural and peri-urban areas of Laos and this is probably due to the differences in environmental and hygienic conditions, health service availability and socio-economic status between the two areas. Therefore strategies for healthcare service improvement may also need to differ between the two areas.

## Background

Respiratory illness (RI) is a leading cause of death and disease burden worldwide [1,2] and about 95% of the deaths from RI occur in developing countries [3]. In the Lao PDR (Laos), RI is an important cause of morbidity and mortality, particularly among children, but very little is known about the overall burden of RI. The latest national health survey in 2000 demonstrated that ~ 1% of under-five children had had acute respiratory infection in the two weeks before the survey [4]. No RI surveillance system existed in Laos until recently, when a facility-based surveillance system was established that uses disease reports originating from hospitals and health centres. The capacity of this surveillance system to reflect the actual disease burden in the community may be limited.

Since healthcare seeking behaviors and healthcare utilization practices vary widely from one community to another, health utilization assessments can assist in identifying the extent to which facility-based data reflect the actual burden of disease in a community [5]. In addition, understanding community patterns of healthcare-seeking behavior (for example who in the community are the first points-of-care for villagers with RI) and reporting would improve public health practice in the community. This survey was conducted to provide better direction for Lao public health program and policy planners to improve public health practice, surveillance systems and prevention strategies, and to generate important information to help evaluate ongoing Lao Government health activities.

## Methods

### Study duration and sites

**Figure 1:**
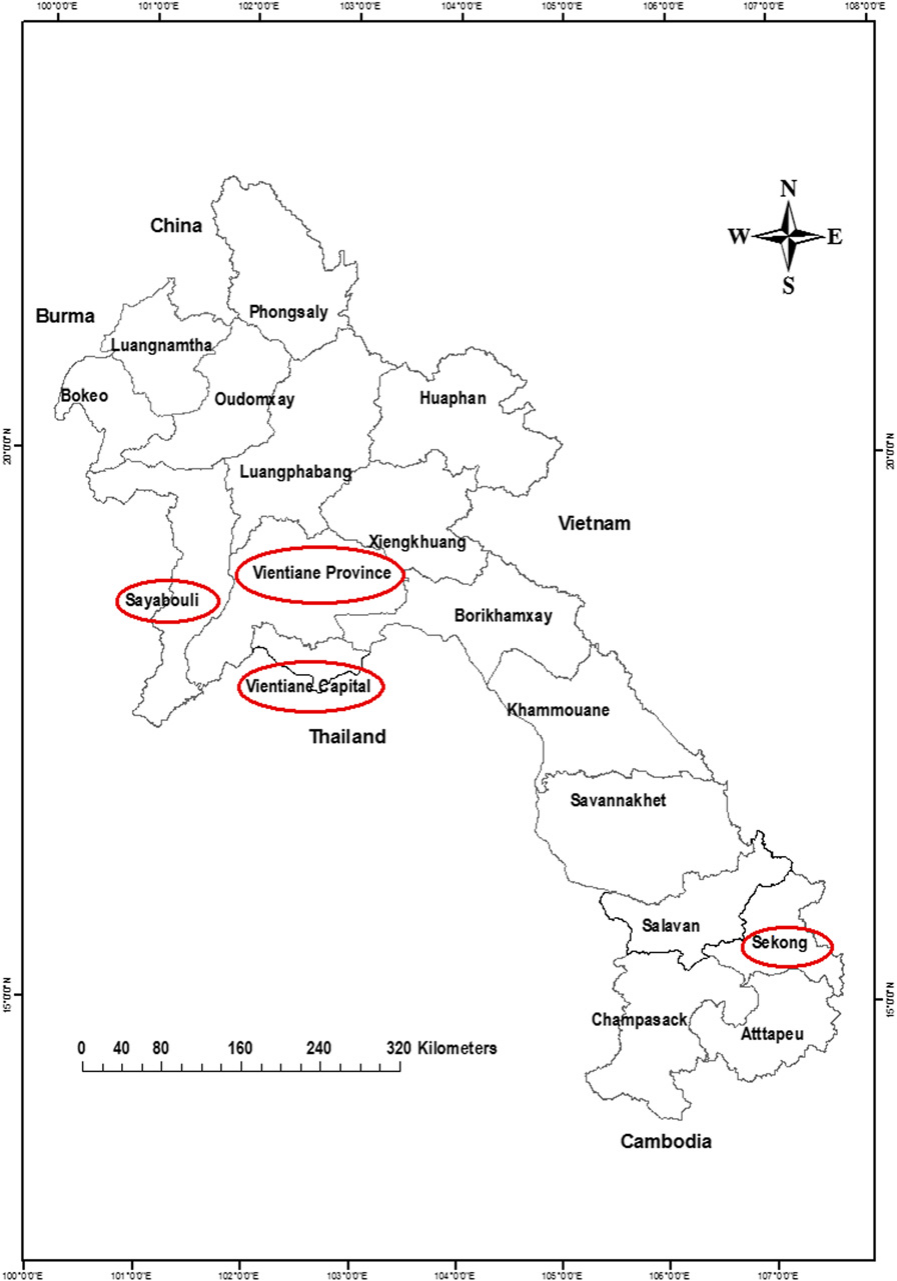
Map of Laos highlighting Vientiane Capital, Vientiane Province, and the study sites of Sayabouli and Sekong Provinces.

The study was conducted between 11 March and 12 April 2009 in peri-urban (Vientiane Capital and Vientiane Province) and rural (Sayabouli and Sekong Provinces) areas (Figure 1). These localities were chosen because they cover the range of peri-urban and rural areas, are sites for the development and piloting of village-based mechanisms to promote human and animal avian influenza surveillance and response, and had had previous avian influenza or influenza-like illness outbreaks. Vientiane Capital and Vientiane Province are located in central Laos, with 9 and 13 districts respectively, comprising both urban and peri-urban areas: one peri-urban district from each was studied. Sayabouli Province is located in northern Laos on the Lao-Thai border, with 10 districts which comprise largely rural areas, with some being very remote and poor. Sekong Province is located in southern Laos on the Vietnam border, with four districts comprising rural areas, and multiple ethnic minority groups [6]. One rural district from each of these provinces was selected for the study. During the study, the climate in Sekong and Xayabouli was relatively cool, while it was less cool in Vientiane Capital and Vientiane Province. The healthcare system in Laos includes four levels: village, district, provincial and central. Village health volunteers (VHVs) (who are usually trained for 2 weeks on how to use basic oral medicines and provide primary healthcare services at their own homes) are the key health providers at the village level. In some villages, health centres are also available, but mostly nurses and medical assistants provide care. At district level, level A hospitals provide care that includes some surgery, while surgery is not undertaken at level B hospitals. The majority of healthcare providers at this level are medical assistants, with a small number of fully qualified doctors. Provincial hospitals provide a higher level of care including surgery and there are more doctors compared than at district level. Central hospitals are tertiary referral hospitals in Vientiane Capital where the majority of the providers are fully qualified doctors and specialists. At the time when the study was conducted, community health insurance schemes were not available at the study sites and these did not become available until the end of 2009. Therefore, people had to pay for their own healthcare throughout the study period.

### Definition of respiratory illness

In order to enable comparisons between assessments, standardized definitions were used. The definition of RI was derived and modified from the WHO case definition for human infections with influenza A (H5N1) virus [2], namely: self-reported new fever with cough and/or a sore-throat in the absence of other diagnoses during the 30 days before the survey. This definition was agreed in consultation with experts from the CDC International Emerging Infections Program and the National Centre for Immunizations and Respiratory Diseases, Influenza Division.

### Sampling procedure and survey

The provinces and districts were purposively sampled as mentioned above, while the villages were randomly selected from the village list of each district. Three villages were selected at random from each district and all households in the village were studied. All members of the households were eligible for the survey. An individual was considered a member of a household if he/she had slept within that compound for at least six of the preceding twelve months. Household members who had died in the past 30 days were also included, as they might have had an episode of RI.

Once written informed consent had been obtained, the recognized heads of households were interviewed. If the household head was not available or was unable to answer the questionnaire, the next most senior adult in the same household was interviewed instead. A pre-designed, structured questionnaire covering socio-demographic information, RI in household members, healthcare-seeking behavior, and household and animal care practices, was used to collect information. The questionnaire was designed in consultation with experts from the CDC International Emerging Infections Program and the National Centre for Immunizations and Respiratory Diseases, Influenza Division. The interviews were conducted in Lao and, when needed, a translator helped with the translation of local dialects.

Sixteen focus group discussions (FGDs) were conducted (eight in each urban and rural area). The participants in the qualitative study were recruited from those who took part in the questionnaire interviews. Eligible participants were selected purposively based on their having at least one family member who had had RI symptoms at least once during last month and their willingness to participate. There were seven to eleven participants per FGD with varying occupations, ages and educational backgrounds. Each FGD lasted approximately 1–1.5 hours and was tape-recorded. Translators were used to conduct FGDs with some ethnic minority women in rural areas. The tapes and notes of the FGDs were transcribed into Lao and then translated into English.

Written informed consent was obtained from all participants before the interviews and FGDs. Ethical clearance for the study was granted by the Ethics Committee of the University of Health Sciences, Ministry of Health of Laos.

### Sample size determination

Assuming that 2.2% of all individuals would have had an episode of RI in the 30 days preceding the assessment [7] with an alpha of 0.05 and estimated precision of 1%, a sample size of 865 households was needed. The calculated sample size was, however, adjusted for a design effect of 2.0 to account for correlations within clusters; therefore we aimed to include a total of 1,752 households.

### Data analysis

Quantitative data were analyzed using Stata v9 (StataCorp, College Station, TX, USA). Comparisons between two groups were made using *Chi-square, Fisher’s Exact, Student-t, and Mann–Whitney U* tests as appropriate. Multiple logistic regression analysis was performed to identify factors associated with RI and RI seeking healthcare. All variables with *P*-values < 0.05 in univariate analysis were included in the multiple logistic regression model and this was processed using the backward stepwise method. Qualitative data were analyzed using a participatory process in parallel with software-based analysis using MAXQED. Transcripts were read and reread and open codes were developed to capture emerging themes in the data. Then the key themes and concepts from the English transcripts were identified. The themes were systematically catalogued across all focus groups. A description of how the themes were described and explained by the FGD participants was then made. Finally, the themes were compared across major subgroups of participants.

## Results

**Figure 2:**
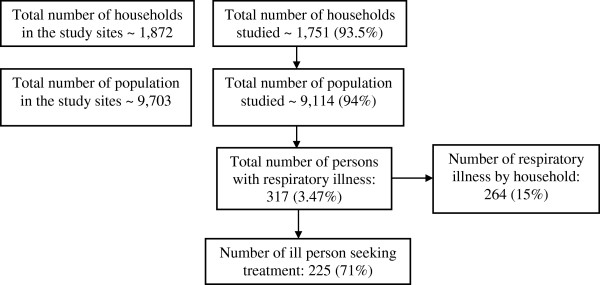
Flow chart of study population.

Of all 1,872 households and 9,703 villagers in the target study sites, 1,751 (93.5%) and 9,114 (94%) respectively were included in the study (Figure 2). The reasons for non-recruitment were that the household members were not in the villages during the survey or had moved away.

### Socio-economic and demographic characteristics of study households and population

Of the study population, 50% were males, 72% were from peri-urban areas, and 59% were Lao Loom (Table 1). The median (IQR) age of all study villagers was 23 (11–39) years and this was significantly lower in rural [17 (8–32)] compared with peri-urban [25 (13–41)] areas (p<0.001). Thirty-four percent of the study population were children ≤15 years and 10% were aged <5 years. The overall median (IQR) highest number of schooling years in the households was 10 (5–11) and this was statistically lower in the rural [5 (2–5)] than in the peri-urban [11 (8–11)] areas (p<0.001). Overall, 62% of the study population were classified as poor and the proportion of the study population classified as poor was significantly higher in rural than in peri-urban areas (p<0.001) (Table 1). The overall mean (95% CI) number of bedrooms in the households was 2.2 (2.1-2.3) and 98% of all households used wood/charcoal stoves for cooking. The mean (95% CI) number of household members who smoked inside the house was 1.5 (1.4-1.6).

**Table 1:** **Socio-demographic characteristics of the study population**^**†**^

** Variable**	**Total**	**Peri-urban (VT Capital & Province)**	**Rural (Xekong & Xayabuli Provinces)**	**p *- *value**
Households studied	1,751 (100)	1,330 (76)	421 (24)	-
People studied	9,114 (100)	6,532 (72)	2,582 (28)	-
Villagers who died during the 30 days before interview	7 (0.07)	5 (0.076)	2 (0.077)	0.99
Female	4,571 (50)	3,243 (50)	1,328 (51)	0.12
Median (IQR) age (years)	23 (11–39)	25 (13–41)	17 (8–32)	<0.001
Adult > 15 years	6,021 (66)	4,643 (71)	1,378 (53)	<0.001
Children ≤ 15 years	3,093 (34)	1,889 (29)	1,204 (47)	
Children < 5 years	938 (10)	554 (8)	384 (15)	<0.001
Median (IQR) highest schooling years in household	10 (5–11)	11 (8–11)	5 (2–5)	<0.001
Ethnicity				
- Lao Loom	5,346 (59)	5,334 (82)	12 (0.5)	<0.001
- Hmong	1,168 (13)	1,166 (18)	2 (0.08)	
- Prai	1,714 (19)	0	1,714 (66)	
- Alak	332 (4)	0	332 (13)	
- Taliang	254 (3)	0	254 (10)	
- Dakkang	268 (3)	1 (0.01)	267 (10)	
- Mixed	5 (0.1)	0	5 (0.2)	
- Others	27 (0.3)	1 (0.01)	26 (1)	
Wealth index*				
- Poorest	2,098 (23)	29 (0.5)	1,797 (70)	<0.001
- Second	1,824 (20)	1,043 (16)	781 (30)	
- Middle	1,730 (19)	1,825 (28)	1 (0.04)	
- Fourth	1,735 (19)	1,815 (28)	3 (0.1)	
- Richest	1,727 (19)	1,820 (28)	0	

^†^Data shown as number (%) unless indicated.

* Principal components analysis was performed by using information on the ownership of household goods and amenities (assets) to assign weights to each household asset and obtain wealth scores for each household in the sample. The assets used in these calculations were as follows: electricity, clock, radio, electric fan, mattress, black and white television, color television, CD/VCD player, water pump, bed, DVD player, satellite, mobile telephone, telephone, refrigerator, air conditioner, washing machine, sofa, watch, bicycle, oxcart, motorbike, tractor, tuk-tuk, car/truck, engine boat, type of poorest quintile to the richest quintile, based on the wealth scores of households they were living in. The wealth index is assumed to capture the underlying long-term wealth through information on the household assets, and is intended to produce a ranking of households by wealth, from poorest to richest. The wealth index does not provide information on absolute poverty, current income or expenditure levels, and the wealth scores calculated are applicable for only the particular data set they are based on. Further information on the construction of the wealth index can be found in Rutstein and Johnson, 2004, and Filmer and Pritchett, 2001.

### Respiratory illness burden and disease severity, and factors associated with RI

The overall proportions of persons and households with RI during the 30 days before interview were 3.5% (317/9,114) and 15% (264/1,751), respectively (Table 2). The percentage of persons who had had RI was significantly higher in rural (6.2%) than in peri-urban (2.4%) areas (p<0.001) (Table 1). This figure corresponds to 10.6% (91/855) for Sekong, 4.0% (70/1,727) for Xayabouli, 2.8% (100/3,611) for Vientiane Capital, and 1.9% (56/2,921) for Vientiane Province (p<0.001). The proportion of persons with RI was significantly lower in Lao Loom than in non-Lao Loom groups [143/5,346 (2.7%) vs 174/3,768 (4.6%), *P*<0.001]. The proportion of the persons with RI among specific ethnic groups was 12.6% (42/332) for Alak, 10.1% (27/268) for Dakkang, 8.6% (22/254) for Taliang, 4.1% (70/1,741) for Prai, 2.7% (143/5,346) for Lao Loom, and 1.1% (13/1,168) for Hmong (p<0.001). The overall median (IQR) age of ill people was 17 (5 – 40) years: 48.5% of them were children ≤15 years and 24% were children under five. The percentage of people who had suffered from RI was 2.7% (168/6,246) among persons aged ≥15 years, 3.7% (72/1,930) for those aged 5 – 14 years, and 8.2% (77/938) for children < 5 years (p<0.001). There was no difference in RI frequency between males [3.7% (168/4,543) and females [3.3% (149/4,571)] (p=0.25).

**Table 2:** **Information on RI and treatment seeking among the study population**^**†**^

** Variable**	**Total**	**Peri-urban (VT Capital & Province)**	**Rural (Xekong & Xayabuli Provinces)**	**p-value**
Households with RI	264 (15)	133 (10)	131 (31)	<0.001
Persons with RI	317 (3.5)	156 (2.4)	161 (6.2)	<0.001
Median (IQR) age (years) of persons with RI (n = 317)	17 (5 – 40)	13 (4 – 37)	20 (5 – 41)	0.23
Age group of persons with RI				
- Adult > 15 years	163 (51.5)	70 (45)	93 (58)	0.02
- Children ≤ 15 years	154 (48.5)	86 (55)	68 (42)	
- Children < 5 years	77 (24)	41 (26)	36 (22)	0.42
- Elderly (> 65 years)	5 (2)	5 (3)	5 (3)	0.96
RI by ethnic groups				
- Lao Loom	143 (45)	143 (92)	0	<0.001
- Hmong	13 (4)	13 (8)	0	
- Prai	70 (22)	0	70 (43)	
- Alak	42 (13)	0	42 (26)	
- Taliang	22 (7)	0	22 (14)	
- Dakkang	27 (8.5)	0	27 (17)	
Persons with RI who sought treatment				
- Overall	225 (71)	147 (94)	78 (48)	<0.001
- Lao Loom	136 (60)	136 (92)	0	<0.001
- Hmong	11 (5)	11 (8)	0	
- Prai	27 (12)	0	27 (35)	
- Alak	31 (14)	0	31 (40)	
- Taliang	9 (4)	0	9 (11)	
- Dakkang	11 (5)	0	11 (14)	

^**†**^Data shown as number (%) unless indicated.

Among those who were reported to have been ill, 219 (69%), 77 (24%), and 21 (7%) of illnesses were classified as mild (no disturbance in daily activities), moderate (some daily activities limited), and severe (cannot work or do daily activities), respectively, and 8 (3.5%) were admitted to hospital, with a median (IQR) number of days in hospital of 4.0 (2.5-4.5). The overall median (IQR) duration of the illness was 4 (3–7) days. Fever (100%), cough (90%) and sore-throat (83%) were the main symptoms reported, followed by runny nose (70%), headache (65%), muscle ache (58%), difficult or fast breathing (42%), chills (30%), wheezing (21%), and nausea or vomiting (18.5%) (Table 3).

**Table 3:** **Symptoms and signs by level of illness**^†^

** Symptoms and signs**	**Level of illness**^**§**^			**p-value**
	**Mild**	**Moderate**	**Severe**	
	**n = 219**	**n = 77**	**n = 21**	
Fever	219/219 (100)	76/76 (100)	21/21 (100)	1.00
Chills	48/212 (22.6)	29/76 (38.1)	16/21 (76.1)	<0.001
Headache	119/191 (62.3)	53/71 (74.6)	13/21 (61.9)	0.16
Sore throat	168/199 (84.4)	56/72 (77.7)	18/21 (85.7)	0.41^*^
Runny nose	165/217 (76.04)	40/77 (51.9)	16/21 (76.1)	<0.001
Cough	192/219 (87.6)	72/77 (93.5)	20/21 (95.2)	0.32^*^
Muscle aches	111/188 (59.04)	37/71 (52.1)	16/21 (76.1)	0.14
Difficult or fast breathing	78/208 (37.5)	32/75 (42.6)	17/21 (80.9)	0.001
Wheezing	39/211 (18.4)	15/75 (20)	10/21 (47.6)	0.01^*^
Decreased daily activity	64/207 (30.9)	39/75 (52)	15/20 (75)	<0.001
Nausea or vomiting	32/216 (14.8)	21/75 (28)	5/21 (23.8)	0.03^*^

^†^Data shown as number (%) unless indicated.

* Fisher’s exact test.

^§^ Symptoms and signs were classified as mild if there was no disturbance in people’s daily activities, as moderate if some daily activities were limited, and as severe if they were not able to work or do daily activities.

The disease severity was not significantly different between children and adults (p=0.35). With the exception of muscle pain, which was significantly more frequent in adults (66%) compared to children (49%) (p=0.005), other symptoms and signs were similar between the groups. The median (IQR) number of days of illness was also significantly greater in adults than in children [5 (3–7) vs 4 (3–7), *P*=0.02].

Of 229 adults with RI, 143 (62%) had been absent from their daily work during their illness and 65% of their family members had also been absent from their daily activities in order to look after them.

Factors associated with RI are shown in Table 4. In a multiple logistic regression analysis, factors significantly related to RI were children <5 years, people living in Sayabouli and Sekong, and a very smoky environment inside the house (Table 5).

**Table 4:** Factors associated with respiratory infection (RI)

**Variable**	** RI**		**p-value**	**OR**	**95% CI**
	**Yes n (%)**	** No n (%)**			
	** N = 317**	**N = 8,797**			
Sex: - Male	168 (53)	4,375 (50)		1	
- Female	149 (47)	4,422 (50)	0.25	0.9	0.7 - 1.1
Ethnic group: - Lao Loom	143 (45)	5,203 (59)		1	
- Hmong	13 (4)	1,155 (13)	<0.001	2.4	1.9 - 3.0
- Other ethnic groups	161 (51)	2,439 (28)	0.002	0.4	0.2 - 0.7
Age group (years): < 5	77 (24)	861 (10)	<0.001	3.2	2.4 - 4.3
5–14	72 (23)	1,858 (21)	0.01	1.4	1.0 - 1.8
≥15	168 (53)	6,078 (69)		1	
Location: - Vientiane Capital	100 (31)	3,511 (40)		1	
- Vientiane Province	56 (18)	2,865 (33)	0.03	0.6	0.4 - 0.9
- Sayabouli	70 (22)	1,657 (19)	0.01	1.4	1.1 - 2.0
- Sekong	91 (29)	764 (7)	<0.001	4.2	3.1 - 5.6
Wealth index: - Low	209 (66)	4,821 (55)	0.08	1.3	0.9 - 1.8
- Moderate	61 (19)	2,526 (29)	0.13	0.7	0.5 - 1.1
- High	47 (15)	1,450 (16)		1	
Household head education level:					
- Primary school	136 (43)	2,567 (29)	<0.001	2.2	1.5 - 3.3
- Secondary school	146 (46)	4,718 (54)	0.12	1.3	0.9 - 1.9
- College/university	35 (11)	1,512 (17)		1	
Smoky environment inside house:					
- No stove inside	77 (24)	3,458 (39)	0.01	0.6	0.4 - 0.9
- No smoke	44 (14)	1,249 (14)		1	
- Some smoke	92 (29)	2,736 (31)	0.80	0.9	0.6 - 1.3
- Very smoky	104 (33)	1,354 (15)	<0.001	2.1	1.5 - 3.1
Animal slaughtering in the past 30 days in household:					
- No	238 (75)	6,547 (74)		1	
- Yes (without protection)	8 (3)	169 (2)	0.47	1.3	0.6 - 2.6
- Yes (with protection)	71 (22)	2,081 (23)	0.64	0.9	0.7 - 1.2
Butchering and cooking animal in the past 30 days in household:					
- No	111 (35)	2,278 (26)		1	
- Yes (without protection)	4 (1)	114 (1)	0.52	0.7	0.2 - 1.9
- Yes (with protection)	202 (64)	6,405 (73)	<0.001	0.6	0.5 - 0.8
Animal died in the past 30 days in household:					
- No animal in household	49 (15)	1,722 (20)		1	
- No animal died	232 (73)	6,554 (74)	0.17	1.2	0.9 - 1.7
- Animal died	36 (11)	521 (6)	<0.001	2.4	1.5 - 3.7

**Table 5:** Multiple logistic regression analysis of factors associated with RI

**Variable**	**OR**	**95% CI**	**p-value**
Being ethnic Hmong	0.4	0.2 - 0.8	0.01
Being others ethnicities	0.8	0.1 – 4.6	0.84
Aged < 5 years	2.7	2.1 - 3.7	<0.001
Aged 5 – 14 years	1.2	0.9 - 1.7	0.08
Living in Vientiane Province	0.9	0.6 - 1.3	0.61
Living in Sayabouli	1.6	1.1 - 2.6	0.03
Living in Sekong	4.1	2.5 - 6.8	<0.001
Education of household head of primary school	0.7	0.5 - 1.1	0.07
No stove inside the house	0.5	0.4 – 0.8	0.004
Very smoky environment in the house	1.8	1.3 - 2.7	<0.001
Butchering and cooking animal in the past 30 days in household with protection	1.2	0.8 - 1.5	0.28
Animal died in household in the past 30 days	1.01	0.6 - 1.7	0.95

### Treatment seeking behavior and preference, and factors associated with RI seeking healthcare

Of all villagers with RI, 225 (71%) sought treatment [94% in peri-urban and 48% in rural areas (p<0.001)] (Table 2). The percentage of persons with RI who sought treatment among each ethnic group was 95% (136/143) for Lao Loom, 85% (11/13) for Hmong, 74% (31/42) for Alak, 41% (9/22) for Taliang, 41% (11/27) for Dakkang, and 39% (27/70) for Prai (p<0.001). Sick persons in Vientiane Capital [98% (98/100)] and in Vientiane Province [87.5% (49/56)] sought treatment more often than those from Sekong [56% (51/91)] and Xayabouli [39% (27/70)], (p<0.001).

The first treatment places/health providers chosen by these people were private clinics (29%), health centres (19.5%), pharmacies (15%), village health volunteers (15%), district hospitals (5%), central hospitals (4%), provincial hospitals (3.5%), and mobile clinics (1.3%). A small proportion (1.4%) of these people went to see traditional or spiritual healers as the first choice for their illnesses. Lao Loom were likely to seek treatment at private clinics (47%) and pharmacies (21%) while non-Lao Loom preferred to go to health centres (39%) and to see village health volunteers (37%) (p<0.001).

The most frequent reasons given for selecting these places/health providers as the first choice were that: it was close to their house (70%); they had previously had good experiences at those facilities/health providers (36%); it was cheap (29%); it provided the appropriate service for their illness (25%); it offered a good quality of care (19%); and that waiting times were short at the facility (19%).

The median (IQR) traveling time, regardless of transportation mode, from patients’ homes to health facilities was 5 (4.5-20) minutes. The most common modes of transport used by sick people to get to the health facilities were walking (44.8%) and motorcycle (43.9%). Eighty-two percent (260/317) of the people who sought treatment were prescribed medication and were able to afford the treatment, while 16% of them could not afford the medicines prescribed by the healthcare providers. The median (IQR) cost of care during their illnesses was LAK 20,000 (10,000-60,000) or ~ 2.5 (1.25 – 7.5) US$.

Factors associated with RI treatment-seeking are shown in Table 6. In a multi-variate analysis, only peri-urban residence was independently correlated with seeking care for RI (OR = 17.9; 95% CI = 8.4 – 37.9, p<0.001) (Table 7).

**Table 6:** Factors associated with respiratory infection (RI) healthcare seeking

**Variable**	** Seeking care**		***P*-value**	**OR**	**95% CI**
	**Yes n (%)**	**No n (%)**			
	** N = 225**	** N = 92**			
Male	119 (53)	49 (53)	0.95	1	
Female	106 (47)	43 (47)		1.01	0.6 - 1.6
Lao Loom	136 (60)	7 (8)	<0.001	20.6	9.1 – 46.9
Hmong	11 (5)	2 (2)	0.02	5.8	1.2 – 27.2
Others	78 (35)	83 (90)		1	
Aged < 5	56 (25)	21 (23)	0.55	1.2	0.6 - 2.2
Age 5–14	53 (23)	19 (21)	0.47	1.2	0.6 - 2.3
Age ≥ 15	116 (52)	52 (56)		1	
Vientiane Capital	98 (43)	2 (2)	<0.001	38.4	8.9 – 165.4
Vientiane Province	49 (22)	7 (8)	<0.001	5.4	2.2 – 13.4
Sayabouli	27 (12)	43 (47)	0.02	0.5	0.2 - 0.9
Sekong	51 (23)	40 (43)		1	
Wealth index:					
- Low	121 (54)	88 (96)		1	
- Moderate	60 (27)	1 (1)	<0.001	43.6	5.9 – 320.8
- High	44 (19)	3 (3)	<0.001	10.6	3.2 – 35.5
Education level of head of household:					
- Primary school	79 (35)	57 (62)		1	
- Secondary school	113 (50)	33 (36)	0.001	2.4	1.5 – 4.1
- College/university	33 (15)	2 (2)	0.001	11.9	2.7 – 51.6
Disease severity:					
- Mild	161 (72)	58 (63)		1	
- Moderate	50 (22)	27 (29)	0.15	0.6	0.3 - 1.2
- Severe	14 (6)	7 (8)	0.50	0.7	0.3 - 1.8

**Table 7:** Multivariate logistic regression analysis of factors associated with RI healthcare seeking

**Variable**	**OR**	**95% CI**	**p-value**
Being ethnic Hmong	0.5	0.08 - 3.5	0.57
Living in Vientiane Province	0.1	0.02 - 0.9	0.04
Living in Sayabouli	0.007	0.001 - 0.05	<0.001
Living in Sekong	0.01	0.001 - 0.09	<0.001
Residence in peri-urban area	17.9	8.4 – 37.9	<0.001
Family with low wealth index	0.3	0.08 - 1.5	0.18
Household head education level of primary school	8.5	1.1 - 66.1	0.44
Household head education level of secondary school	2.2	0.3 - 14.3	0.39

Among those who did not seek treatment, 38% thought their illnesses were just mild and would be self-limiting, and 33% of them said they were not able to afford the treatment and transportation costs. More than half (59%) of the villagers who did not seek care said they only took rest at home, while 31.5% of them self-medicated at home [55.5% in peri-urban and 29% in rural areas (p<0.001)]. The reasons for not seeking care were not significantly different between Lao Loom and non-Lao Loom ethnicities.

### Comparison of treatment-seeking between peri-urban and rural areas

Fewer sick persons sought care in rural (48%) than peri-urban (94%) areas (p<0.001) as they tended to rest at home more often than those in the urban-areas (63% vs 22%, p=0.04). There were no statistically significant differences in the reasons for not seeking care between the sick people who lived in rural and peri-urban areas.

Sick people who lived in peri-urban areas commonly chose private clinics (44.9%) or pharmacies (22.5%) as the first places of care while those who lived in rural areas frequently chose to consult with the village health volunteer (42.3%) or visit the health centre (41.1%) as their first choice (p<0.001). This difference was due to the fact that there were more private clinics and pharmacies in peri-urban than in rural areas.

Quality of care (26% vs 6%, p<0.001), having good previous experience at the facilities (43.5% vs 5%, p=0.01), familiarity with healthcare providers (16% vs 5%, p=0.01), and short waiting times (28% vs 1%, p<0.001), were reasons given significantly more often in peri-urban than in the rural areas for choosing the first places of treatment. More sick people in rural areas walked to the health facility than those in peri-urban areas (94% vs 17%, p<0.001), but more villagers in the peri-urban areas used motorcycles as their mode of transport when compared with those who lived in rural areas (68% vs 2.4%, p<0.001). The median (IQR) traveling time (in minutes) from the place of residence to the first place of treatment was significantly shorter in rural [5 (3 – 15)] than in peri-urban [5 (5 – 20)] areas (p= 0.01). After seeking care, all patients (100%) in peri-urban areas received medication, while 95% of the patients in rural area did so (p=0.01).

The proportion of sick persons who could not afford treatment was also higher in rural (37%) compared to peri-urban areas (4%) (p<0.001), and the median (IQR) cost of medication paid by the patients was significantly higher in the peri-urban [30,000 (15,000-80,000) LAK or ~3.75 (1.87-10.0) US$] than in the rural areas [10,000 (5,000-30,000) LAK or 1.25 (0.62-3.75) US$] (p<0.001). The percentage of sick persons who smoked cigarettes was significantly higher in the rural compared to the peri-urban areas [78/156 (50%) vs 9/161 (6%), p<0.001)].

### Qualitative data on healthcare-seeking behavior

**Figure 3:**
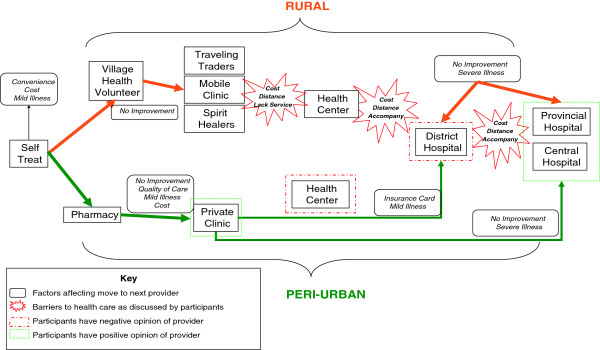
Process mapping of health seeking behaviors in rural and peri-urban areas.

Sixteen focus group discussions (FGDs) were conducted (see above) to explore people’s healthcare-seeking behavior when they had RI. The order of seeking care, and different factors that influenced sick persons as they progressed along a continuum of care, were mapped (Figure 3). In general, all participants first attempted to self-treat any illness at home. Some experiences of self-treatment, such as drinking hot water, taking a sponge bath, self-medication (especially with medical herbs), were documented. More rural than peri-urban participants reported self-treatment. The main reasons given for self-treatment were convenience, lower treatment cost, and the fact that the illness was mild. An example of a comment from one FGD was: *“If there is someone in the household who got sick, if they got sick and they were not severely unwell, they went to buy medicine by themselves. If the illness is severe, they asked someone to buy medicines for them.” (Female, age 18–30 year old; northern Xayabouli province).*

The decision to move from one provider to another (or from self-treatment to seeking some form of treatment) was commonly influenced by the severity of the illness, treatment failure or a lack of improvement, and a lack of medicines. Rural areas were considered by FGD participants as being characterized by constrained access, in terms of infrastructure and distance, together with higher direct and indirect costs of seeking care. Because of these barriers, participants stated that they would seek formal healthcare (e.g. health centre, hospitals) only when they were very ill, unlike their counterparts in urban areas, who seek informal care (e.g. pharmacies) even in cases of very mild illness. Given the higher costs and constrained access, participants in rural areas tended to revert to whatever providers were locally available for treatment of their illness. These participants often used village health volunteers, but also turned to illegal physicians (unregistered providers of medicine and health advice), spiritual healers, traveling salesmen, or self-treatment with herbal medicines in equal numbers. Other than specific cultural beliefs about spiritual healers held by a small number of participants, these ‘informal’ healthcare options were not *preferred* in rural areas, but were used as the only option available because all other options were too expensive and too far away. When people in remote, rural areas say they have “sought treatment”, the type and quality of treatment available is quite different from that available to those in peri-urban or less remote settings. Availability of service providers in rural areas was also much more constrained. Village health volunteers (VHVs) and health centres would sometimes not have the medicines needed; furthermore the equipment and acceptability of care at each place was considered inadequate by some participants.

Cost, distance, and service availability were the primary factors acting as barriers to participants in rural areas accessing formal health services, while cost, service availability and trust were important factors relating to using other types of healthcare providers. Rural participants were less likely to be able to seek any kind of formal healthcare for illness. Clearly those in urban areas have many more options from which to choose, and thus have more ways of comparing different options and choosing where to seek care. They are more likely to receive some form of treatment than those in rural areas. While central hospitals are considered very expensive, most participants in Vientiane municipality would still seek care there if they had a severe illness. Furthermore when cost is similar for two or more options, those in urban areas look to quality and the range of services offered in order to decide which to choose. There are more options and alternative forms of care in urban areas, so that if one option (e.g. central hospital) is too expensive, there are many other possible choices, such as private clinics, provincial hospitals, and district hospitals, especially if the patient has insurance. As a participant in one FGD mentioned: *“Going to the district hospital is more complicated with many steps. I do not like to go there. Seeing doctors at the private clinic is better because it is the same doctor as in the district hospital while the cost is 25,000 LAK ~ 3.12 US$.” (Female, 18–30 years, Vientiane capital)*.

## Discussion

This is the first study on the burden of, and the healthcare-seeking behavior for, RI conducted in Laos. The prevalence of RI in the survey was higher than the prevalence of influenza in rural Thailand (2.2%) [7] and might have been higher if the survey had been carried out during the winter. The Lao national health survey in 2000 revealed that ~ 1% of children under-five had had acute respiratory infection in the two weeks before the survey [4]. Indoor air pollution (i.e. a very smoky environment inside the household) was a significant risk factor for the high prevalence of RI found in the present study. Many households, particularly in rural areas, used firewood/charcoal for cooking inside their houses, some of which had only a single room. The proportion of persons with RI in the present survey was significantly higher in rural than in peri-urban areas, probably due to the younger age of the study population and the poorer socioeconomic, environmental and hygienic conditions in the rural areas, a finding that is consistent with previous studies [8-11]. Childhood immunization coverage, which is usually higher in peri-urban than in rural areas, may also play an important role in reducing the risk of respiratory illness among peri-urban people.

About two-thirds of the people with RI in this study sought treatment, which was a high proportion but similar to that found in previous studies from Sri Lanka, Nigeria, Bangladesh, and Uganda [12-16]. In the Lao national health survey in 2000, only 47% of people who had suffered from any illnesses in the two weeks preceding the survey had sought care [4]. Whether sick people seek care depends on disease severity, distance from home to healthcare facilities, availability of care services, and affordability. Severity of illness could not explain the high percentage of healthcare-seeking in the present study because only 6% of sick persons were classified as having severe disease and the proportion of ill people who sought care was similar among those who had mild, moderate, and severe disease. The high rate of healthcare-seeking of people in the present study might be due to the availability of healthcare services close to their own homes i.e. in their own village. In the national health survey in 2000, the reasons for not seeking care given by the villagers were mild illness, lack of transport, long distance, and cost [4]. The reasons for not seeking care in our survey were inability to afford treatment, transportation, and a perception that their illness was mild. The reasons for not seeking care amongst sick persons in our study were totally different from those in a study from the Dominican Republic, where the most frequent reasons were distrust of the clinic staff, shortage of medicines, and dispersion of the rural population [17].

The results of our exploratory study indicate the important role of self-treatment for RI, particularly in rural areas where the accessibility, availability and affordability of healthcare facilities are limited. The reasons for self-treatment were convenience (proximity), cheapness (affordability), and because the illness was mild (severity). The option of self-treatment as the first choice is usually influenced by the perception of severity. The FGD participants in both peri-urban and rural areas reported that they chose self-medication and self-treatment as first line approach in cases of mild RI. In previous studies it was also found that self-treatment was common when the severity of the illness was perceived to be low [18-20]. In Laos, approximately one third to a half of people self-medicate when they become sick [4,21] – findings that are consistent with those of our study. In Guatemala, about two thirds of children with diarrhea and RIs were self-treated by their guardians or relatives and only one third of illnesses were treated by healthcare providers such as pharmacists, doctors, and staff at health posts and centres [22].

A study in the Dominican Republic demonstrated that the choice of health service utilization among caregivers of children with RI was determined by location (proximity) and cost [15]. Healthcare-seeking behavior in the current survey was very different between peri-urban and rural areas. If there was no improvement following self-treatment, people in peri-urban area preferred to choose private clinics and pharmacies as their next treatment option, while those in rural areas frequently consulted the village health volunteer and visited the health centre. This may be explained by the difference in the availability and proximity of the healthcare facilities between the two areas. In peri-urban areas, private clinics and pharmacies are readily available and nearby when compared with rural areas. In contrast, hardly any private clinics and pharmacies are available in rural areas, apart from the health centre and village health volunteer. It is obvious that Lao villagers prefer to use the closest healthcare facilities because of the convenience and the low travel cost. The national health survey in 2000 also showed that more people in peri-urban and urban areas went to a pharmacy when they were ill compared with those in rural areas [4]. A study in western Nepal revealed that almost half of episodes of childhood illness were treated by pharmacists and in 15% of episodes medicines were purchased from pharmacies without consultation [23].

Severity of illness is an important determinant of treatment-seeking behavior [24,25]. In our study, the decision to move from one healthcare provider to another, or from self-treatment to seeking some form of treatment, was also influenced by severity of illness or treatment failure. Sick persons did not go back to the same healthcare provider or facility which they had originally visited but to other places. In peri-urban areas, people would seek further care in central hospitals, where a better quality of service and modern technology are available. In rural areas, people would like to seek further treatment in the district or provincial hospital, but are constrained from doing so by distance and cost.

The limitations of this study are that the definition of RI used here included only fever with cough and/or sore-throat – symptoms which do not cover many other respiratory illnesses. Another drawback is that the study relied only on self-reporting illness in the previous 30 days, which may be subject to recall and reporting bias. The study was conducted only in a cross-sectional manner in time, and therefore the proportion of RI found in this survey may not be representative of all times of year, especially the winter.

## Conclusions

The burden of RI and related healthcare-seeking behavior are different between rural and peri-urban areas in Laos, and this is probably due to the differences in environmental and hygienic conditions, availability of healthcare services, and socio-economic status between the two areas. Therefore strategies for healthcare service improvement may also need to differ between the two areas. In peri-rural areas, improvement must be focused on private clinics and pharmacies, while it should be focused on health centres and village health volunteers in remote rural areas. Further studies are required, particularly in urban areas and among specific ethnic groups, in order to determine whether there is a difference in the pattern of healthcare-seeking behavior so that specific strategies for the improvement of healthcare services can be established and implemented practically and appropriately.

## Competing interests

The authors declared that they have no competing interests.

## Authors’ contributions

MM, VH, VS developed protocol, collected and analyzed data, and drafted the manuscript. BS, LO, VT, VS, CT, SN, and JP collected data and revised the manuscript. KC and PC analyzed data and revised the manuscript. All authors have read and agreed on the final version of this manuscript.

## Pre-publication history

The pre-publication history for this paper can be accessed here:

http://www.biomedcentral.com/1471-2458/13/444/prepub
